# The Role of Co-chaperones in Synaptic Proteostasis and Neurodegenerative Disease

**DOI:** 10.3389/fnins.2017.00248

**Published:** 2017-05-19

**Authors:** Erica L. Gorenberg, Sreeganga S. Chandra

**Affiliations:** ^1^Interdepartmental Neuroscience Program, Yale UniversityNew Haven, CT, United States; ^2^Department of Neurology, Yale UniversityNew Haven, CT, United States; ^3^Department of Neuroscience, Yale UniversityNew Haven, CT, United States

**Keywords:** Hsp110, HSP70, neurodegeneration, proteostasis, synapse maintenance, endocytosis, exocytosis

## Abstract

Synapses must be preserved throughout an organism's lifespan to allow for normal brain function and behavior. Synapse maintenance is challenging given the long distances between the termini and the cell body, reliance on axonal transport for delivery of newly synthesized presynaptic proteins, and high rates of synaptic vesicle exo- and endocytosis. Hence, synapses rely on efficient proteostasis mechanisms to preserve their structure and function. To this end, the synaptic compartment has specific chaperones to support its functions. Without proper synaptic chaperone activity, local proteostasis imbalances lead to neurotransmission deficits, dismantling of synapses, and neurodegeneration. In this review, we address the roles of four synaptic chaperones in the maintenance of the nerve terminal, as well as their genetic links to neurodegenerative disease. Three of these are Hsp40 co-chaperones (DNAJs): Cysteine String Protein alpha (CSPα; DNAJC5), auxilin (DNAJC6), and Receptor-Mediated Endocytosis 8 (RME-8; DNAJC13). These co-chaperones contain a conserved J domain through which they form a complex with heat shock cognate 70 (Hsc70), enhancing the chaperone's ATPase activity. CSPα is a synaptic vesicle protein known to chaperone the t-SNARE SNAP-25 and the endocytic GTPase dynamin-1, thereby regulating synaptic vesicle exocytosis and endocytosis. Auxilin binds assembled clathrin cages, and through its interactions with Hsc70 leads to the uncoating of clathrin-coated vesicles, a process necessary for the regeneration of synaptic vesicles. RME-8 is a co-chaperone on endosomes and may have a role in clathrin-coated vesicle endocytosis on this organelle. These three co-chaperones maintain client function by preserving folding and assembly to prevent client aggregation, but they do not break down aggregates that have already formed. The fourth synaptic chaperone we will discuss is Heat shock protein 110 (Hsp110), which interacts with Hsc70, DNAJAs, and DNAJBs to constitute a disaggregase. Hsp110-related disaggregase activity is present at the synapse and is known to protect against aggregation of proteins such as α-synuclein. Congruent with their importance in the nervous system, mutations of these co-chaperones lead to familial neurodegenerative disease. CSPα mutations cause adult neuronal ceroid lipofuscinosis, while auxilin mutations result in early-onset Parkinson's disease, demonstrating their significance in preservation of the nervous system.

## Protein folding and chaperones

Proteins must fold into unique three-dimensional structures to fulfill their biological functions. As proteins are inherently structurally dynamic, they are apt to misfold into conformations that prevent their function or lead to toxic aggregates (Bukau et al., 2006; Hartl et al., 2011). This potential for misfolding can be exacerbated by cellular stress, such as that caused by heat shock or hypoxia (Bukau et al., 2006; Hartl et al., 2011). Chaperones are a diverse set of evolutionarily conserved proteins that function to assist in protein folding, assembly, and stability, thereby ensuring homeostasis of the proteome (proteostasis). Chaperones comprise up to 10% of the mass of human cells and unfold, dissociate, refold, or degrade the other 90% of cellular proteins as required (Finka et al., 2016). Chaperones act throughout the lifetime of a given protein, first assisting in folding the nascent polypeptide to its native conformation, then surveilling metastable/misfolded intermediates and disaggregating aggregated proteins, and finally removing terminally aggregated proteins through proteolytic degradation.

Chaperone members are often referred to as stress proteins or heat shock proteins (Hsps) because they are upregulated in conditions of conformational stress. The major chaperone families are classified by molecular weight (Hsp40, Hsp60, Hsp70, Hsp90, Hsp100, and the small Hsp; reviewed in Jee, 2016). All chaperone families, except for the small Hsps, catalyze ATP-dependent processes. The different classes of chaperones perform diverse cellular functions that are coordinated and integrated into a proteostasis network to achieve proper protein and cellular homeostasis. For instance, Hsp70s work in conjunction with Hsp40 co-chaperones (also known as DNAJ or J proteins) and will be discussed in detail below (Otto et al., 2005). The Hsp90s not only support protein folding, but also assist in conformational maturation and maintenance. Hsp90 coordinates with the Hsp70 system through the Hsp70-Hsp90 Organizing Protein (HOP) co-chaperone (Chen and Smith, 1998). Eukaryotic Hsp60 class chaperone TriC is a nano-cage that aids in the folding of cytoskeletal proteins such as actin and tubulin (Frydman et al., 1992). Disaggregases containing the Hsp100 family member Hsp110 are upregulated during proteomic stress when proteins misfold and aggregate. Small Hsps exhibit chaperone-like activity in preventing aggregation of target proteins, maintaining them in a folding–competent state and refolding them independently or in concert with other ATP-dependent chaperones such as Hsp70s (McGreal et al., 2012). For a more comprehensive review on small Hsps, the authors suggest Strauch and Haslbeck (2016).

Chaperone families are compartmentalized within the cell, with unique members in distinct sub-compartments to most efficiently carry out their protein folding functions. Hsp70s and Hsp90s are ubiquitous cytosolic chaperone proteins whose specific localization is determined by their partner Hsp40 co-chaperones. Mitochondria-specific chaperones include mHsp70 and Hsp60 that are involved in importing mitochondrial proteins into the matrix and folding them (Manning-Krieg et al., 1991). Another complement of chaperones is exclusive to the endoplasmic reticulum (ER), the site of synthesis and folding of membrane and secretory proteins. There, ER chaperones such BiP and the ER Hsp70 function to prevent stress from elevated levels of unfolded proteins. Recent papers have highlighted the importance of the chaperones involved in the unfolded protein response at the ER in neuronal proteostasis and neurodegenerative disease (Hetz et al., 2015). Finally, Hsp100s and small Hsps are nearly ubiquitous in terms of cellular localization (Haslbeck and Vierling, 2015; Zuo et al., 2016).

## Unique requirements for synaptic proteostasis

Synapses are specialized junctions that connect neurons into circuits. Synapses must be maintained throughout an organism's life for appropriate brain function and behavior. Chemical synapses consist of a presynaptic compartment from which neurotransmitter is released into a synaptic cleft, juxtaposed to a postsynaptic compartment, with receptors for the corresponding neurotransmitters. The postsynaptic neuron transduces neurotransmitter signals and propagates them as action potentials. Synaptic transmission is noted for its speed (on the order of milliseconds), precision, and spatial control. Synaptic functions require a large complement of specialized proteins, such as neurotransmitter receptors, synaptic vesicle exo- and endocytosis proteins, and organizers of the active zone and post synaptic density, all of which are essential for neurotransmission. Synaptic proteins are under constant proteostatic stress, as they execute reactions that require precise conformational changes and protein-protein interactions at high frequencies. It has also been noted that synapses are enriched in proteins susceptible to misfolding and aggregation, such as α-synuclein, tau, and amyloid-β, all of which are linked to neurodegenerative diseases (Schubert et al., 1991; Fortin et al., 2004; Kramer and Schulz-Schaeffer, 2007; Greten-Harrison et al., 2010; Schulz-Schaeffer, 2010). In addition, synapses can be at great distances from the neuronal cell body, where the majority of proteins are synthesized and properly folded. Though there is strong evidence that a small subset of proteins is locally translated at postsynaptic spines (Weiler et al., 2004) and similar emerging data for the presynaptic termini (Shigeoka et al., 2016; Younts et al., 2016), most newly-synthesized proteins must be transported from the cell body along the full length of the axon. The slow rates of axonal transport are not ideal for replacing misfolded or dysfunctional synaptic proteins on demand. Due to the sustained nature of neurotransmission, the synaptic proteome is perilously prone to protein misfolding for the reasons stated above, yet perseveration of the proper structure and function of synaptic proteins is fundamentally important to the health and survival of neurons.

To maintain their specialized structure and function, neurons possess dedicated synapse-specific proteostasis machinery localized to pre- and post-synaptic compartments, of which chaperones are an integral component. In fact, 4% of the synaptic vesicle compartment proteome is composed of chaperones (Phillips et al., 2005; Burré et al., 2006; Table 1). The chaperones found at the synapse include Hsp60, Hsp70, and Hsp90 members: Hsc71, Hsp70, Hsp70-4, Hsc70, Hspa5, Hsp8, Hspa4, Hspa4l, inducible and constitutive Hsp90, as well as Hsp105/110, and heat shock factor binding protein 1 (Phillips et al., 2005; Burré et al., 2006; Zhang et al., 2012; Zhang and Chandra, 2014). The synaptic chaperone complement also includes Hsp40/DNAJ proteins such as DNAJC6 (auxilin), DNAJC5 (CSPα), cognate of Hsp40-3, DNAJA1 homologs, DNAJB1 and DNAJB2 (Stetler et al., 2010; Table 1). Autophagy is also a mechanism employed at synapses for the removal of misfolded proteins (Hara et al., 2006; Komatsu et al., 2006; Ariosa and Klionsky, 2016). As such, atg3, Atg16L1, LC3B, and Rab33B, which are essential for autophagy, have been found at synaptic termini (Binotti et al., 2015; Soukup et al., 2016). In this review, we will focus on Hsc70/Hsp40 chaperone complexes that are enriched at the synapse, their modes of action, and the mechanisms by which mutations in these genes cause neurodegenerative disease.

**Table 1:** **Summary of chaperones identified at the synapse**.

**Synaptic chaperone**	**Method of identification**	**Reference**
**HSP70s AND 90s**		
Hsc71	1-D SDS; PPF extraction	Phillips et al., 2005; Burré et al., 2006
Hsp70	DIGE; PPF extraction	Phillips et al., 2005; Zhang et al., 2012
Hsc70	DIGE/iTRAQ; PPF extraction	Phillips et al., 2005; Zhang et al., 2012
Hsp90	DIGE	Zhang et al., 2012
Hsp70-4	iTRAQ	Zhang et al., 2012
Hsp84	dSDS	Burré et al., 2006
Hspa5	DIGE	Zhang et al., 2012
HspA4/A4L	iTRAQ	Zhang et al., 2012
Hsp8	dSDS; PPF extraction	Phillips et al., 2005; Burré et al., 2006
**HSP40s/DNAJs**		
DNAJC6 (auxilin)	1-D SDS	Burré et al., 2006
DNACJ5 (CSPα)	iTRAQ	Takamori et al., 2006; Zhang et al., 2012
DNAJ homologs	PPF extraction	Phillips et al., 2005
Hsp40-3 cognate	PPF extraction	Phillips et al., 2005
DNAJA	DIGE/iTRAQ	Zhang et al., 2012
Hsp40 (DNAJB1)	Western Blot	Suzuki et al., 1999
**HSP60**		
Chaperonin TriC	DIGE	Zhang et al., 2012
**HSP100s**		
Hsp105/110	iTRAQ	Zhang et al., 2012
**OTHER CO-CHAPERONES**		
HSF binding protein 1	PPF extraction	Phillips et al., 2005
Calnexin	1-D SDS	Burré et al., 2006
CCT family	1-D SDS	Burré et al., 2006
FLJ10737 cognate	PPF extraction	Phillips et al., 2005
HOP	DIGE	Zhang et al., 2012
HIP	DIGE/iTRAQ	Zhang et al., 2012

*Abbreviations: 1-D SDS, One dimensional sodium dodecyl sulfate electrophoresis; PPF, post-synaptic particle fraction; DIGE, 2D fluorescence Difference Gel Electrophoresis; iTRAQ, Isobaric Tag for Relative and Absolute Quantitation*.

## Hsp70, Hsc70, Hsp40 proteins

Stress-inducible Hsp70s and their constitutive relative, heat shock cognate 70 (Hsc70) are first responders to cellular stress and cases of misfolded protein accumulation. In the nervous system, induction of Hsp70 by stress is weak, therefore Hsc70 is the main Hsp70-class chaperone (Pardue et al., 1992; Marcuccilli et al., 1996; Kaarniranta et al., 2002; Batulan et al., 2003).

As the chief cytosolic chaperones, Hsp70/Hsc70s are involved in executing myriad ATP-dependent protein folding reactions. Hsp70/Hsc70s consist of a 44-kD, N-terminal nucleotide binding domain (NBD) that allows them to bind and hydrolyze ATP. The NBD is connected via a hydrophobic linker to a 25 kD substrate binding domain, that binds preferentially to hydrophobic sequences (Flaherty et al., 1990; Blond-Elguindi et al., 1993). Hsp70s can also contain an additional C-terminal domain that allows for interactions with their Hsp40 co-chaperones and refolding of specific substrates (Radons, 2016).

**Figure 1:**
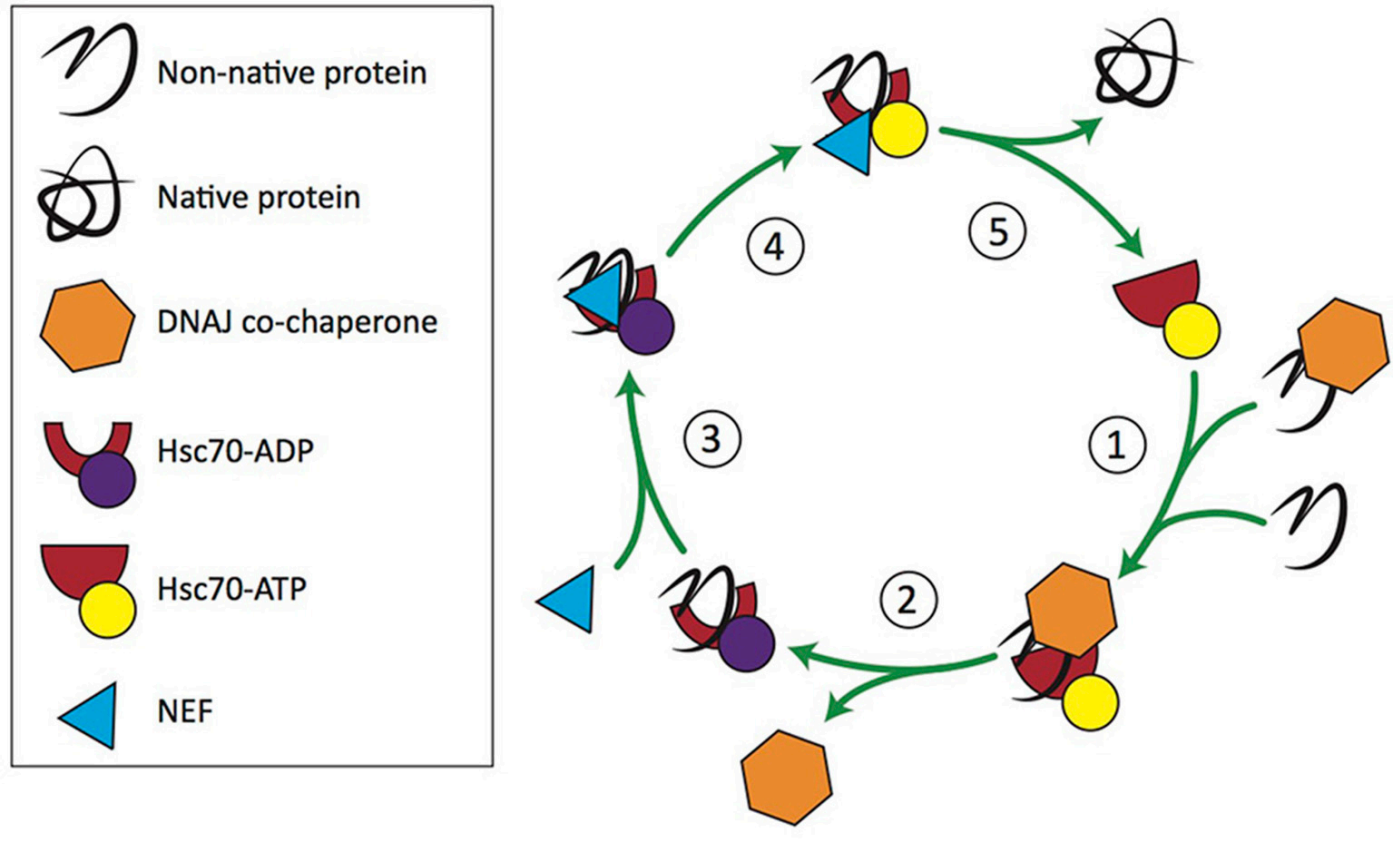
**Hsc70/DNAJ co-chaperone cycle**. 1. Free clients or those recruited by DNAJ bind Hsc70-ATP. 2. DNAJ stimulates Hsc70's ATPase activity. 3. An Hsc70 nucleotide exchange factor (NEF) binds Hsc70-ADP. 4. NEF stimulates ADP-ATP exchange. 5. Hsc70-ATP releases the client and enters a new round of chaperone activity.

Hsc70 recognizes five- to seven-amino acid segments in protein substrates (also known as clients) that are enriched in hydrophobic residues likely to misfold, or in regions that are susceptible to β aggregation (Flynn et al., 1991; Blond-Elguindi et al., 1993; Behnke et al., 2016). Hsc70 is promiscuous in its binding to clients but has a nucleotide-dependent conformational cycle that regulates its association with clients and its chaperone activity (Figure 1; reviewed in Mayer, 2013). Hsc70 has rapid client binding-release activity when bound to ATP, and slow, inefficient binding and release rates when bound to ADP (Nollen et al., 2000; Kampinga and Craig, 2010). Hsc70 is dependent on its Hsp40 co-chaperones to accelerate the rate of ATP hydrolysis to facilitate client binding (discussed further below), and on NEFs to accelerate ADP-ATP exchange for client release (Bracher and Verghese, 2015). NEFs, such as Hsp110, are responsible for restoring Hsc70 to its active form. Each combination of Hsp40 and NEF is required at a different stoichiometric ratio with Hsc70 to stimulate maximum chaperone activity (Rauch and Gestwicki, 2014). For a comprehensive review of the Hsc70 NEFs, see Bracher and Verghese (2015).

Hsp40 co-chaperones (traditionally called DNAJ proteins or J proteins after *DnaJ* in *E. coli*) are crucial players in the Hsc70 conformational cycle (Rassow et al., 1995). Hsp40s stimulate Hsp70 ATP hydrolysis, the rate-limiting step, resulting in as much as a 150,000-fold increase in Hsp70's ATPase activity at 5°C (Russell et al., 1999). In addition, Hsp40s play crucial roles in targeting Hsp70s to distinct sub-compartments and binding clients, thus increasing the regional diversity and specificity of Hsc70/Hsp40 clients. Humans, for example, have 11 Hsp70s that interact with 41 Hsp40s, thereby generating a large cohort of potential combinatorial chaperone complexes (Kampinga and Craig, 2010). Hsp40s are broadly expressed throughout the nervous system, and within neurons in sub-compartments ranging from the cytosol and nucleus, to the ER, to the mitochondria (Finka et al., 2016). For instance, Hsp40 is the postsynaptic co-chaperone, localized to dendritic spines and believed to play a role in synaptic plasticity through its interactions with Hsp70 (Suzuki et al., 1999), while CSPα is a Hsp40 co-chaperone located at the presynaptic terminal.

**Figure 2:**
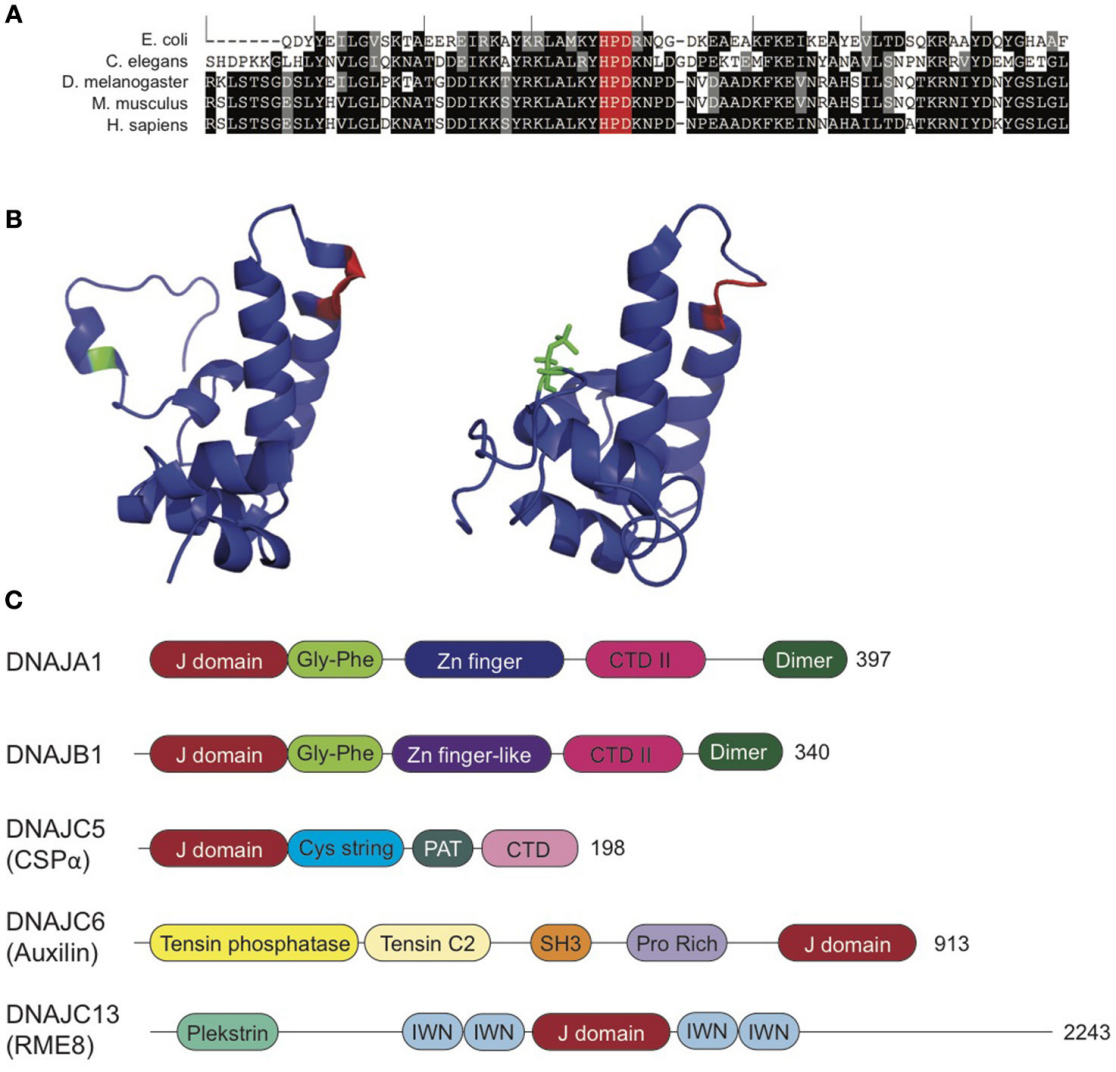
**Synaptic Co-Chaperones. (A)** DNAJ proteins exhibit high homology within the J domain. J domains from *E. coli* DNAJ, *C. elegans* DNAJ14 and *D. melanogaster, M. musculus*, and *H. sapiens* DNAJC5. Black indicates identical residue. Gray indicates amino acids with conserved properties. The conserved HPD motif is highlighted in red. Sequence alignments generated using T Coffee (Notredame et al., 2000; Di Tommaso et al., 2011). **(B)** CSPα J domain exhibits conformational shift with phosphorylation at serine 10 (green). Unphosphorylated (left, PDB: 2N05) and phosphorylated (right; PDB: 2N04) CSPα J domain. HPD motif is highlighted in red. See, Patel et al. (2016) for further details. **(C)** Domain organization of J co-chaperones reviewed in this article: DNAJA1 (PDB: 2M6Y), DNAJB1 (Kampinga and Craig, 2010), DNAJC5 (CSPα; PDB: 2N04 and 2NO5), DNAJC6 (auxilin; PDB: 3N0A), and DNAJC13 (Zhang et al., 2001). Not to scale. See also, Kampinga and Craig (2010). CTD, C-terminal domain; IWN, 90-amino acid conserved motif; PAT, palmitoylacyltransferase recognition region.

Structurally, Hsp40s are defined by their conserved J domain and typically have large variation in the rest of the protein (Figure 2A). Classical Hsp40s have both a J domain as well as a zinc finger domain that regulates client protein binding. The J domain of Hsp40 proteins consists of a 70-amino acid sequence with four alpha helices (Figure 2B), which is conserved in co-chaperones from *E. coli* to humans (Tobaben et al., 2003). Within the J domain, the histidine, proline, aspartic acid (HPD) motif, which falls between the second and third alpha helices, is highly conserved, as it is necessary for stimulation of Hsp70 ATPase activity (Jiang et al., 2007). Despite the conservation of the J domain, Hsp40s range in size from 18 to 520 kD (Koutras and Braun, 2014; Fontaine et al., 2016). The structural diversity outside of the J domain allows the Hsp40 co-chaperones to provide specificity to Hsc70 activity, as these regions function in the recruitment of clients to Hsc70 (Behnke et al., 2016). Hence, Hsp40s are drivers of Hsp70 functional diversity.

The Hsp40/DNAJ proteins are grouped into three classes: A, B, and C, based on motifs and domains present (Hageman and Kampinga, 2009). Class A DNAJ proteins are structurally similar to *E. coli DnaJ* (Goffin and Georgopoulos, 1998) and have an N-terminal J domain, followed by a Gly and Phe-rich region, four repeats of the CxxCxGxG type zinc finger motif and a C-terminal domain, which is now known to bind client proteins. Class B DNAJ proteins also have high homology with the domains of *E. coli* DNAJs, but lack the zinc finger domain. The only conserved feature of members of the DNAJC class is the J domain, but not necessarily in its canonical location at the N-terminus (Kampinga et al., 2009). The greater variation in the DNAJC protein structure provides these proteins with highly specific client interactions as compared to the more nonspecific, promiscuous binding of the A- and B-class DNAJ proteins (Figure 2C).

The Hsc70-DNAJ chaperone complex functions in nascent folding, refolding, disaggregation, and degradation of a wide range of proteins (Fontaine et al., 2016). There is increasing evidence for DNAJ dysfunction as a cause for disease, as seven J chaperones have mutations linked to neurological disorders. Besides the three that will be discussed below, *DNAJC19* (TIM14) and *DNAJC29* (sacsin) are linked to forms of ataxia, *DNAJB2* (HSJ1) is linked with distal motor neuropathy, *DNAJC12* is associated with phenylketonuria-related neurodevelopmental deficits, and *DNAJB6* (Mrj) is linked with limb-girdle muscular dystrophy type 1D (Koutras and Braun, 2014; Anikster et al., 2017). *DNAJB2* was also recently identified as a cause of spinomuscular atrophy and Parkinsonism (Sanchez et al., 2016). In this review, we will focus on synaptic Hsc70 chaperone complexes with DNAJC proteins CSPα, auxilin, and RME-8, as well as DNAJA1 and B1 in their interactions with Hsp110.

## DNAJC5: cysteine string protein α

CSP was discovered in *Drosophila melanogaster* in a screen of antibodies labeling the nerve terminal (Zinsmaier et al., 1990) and was then shown to be expressed mainly in the brain and retina. Immunohistochemistry analysis revealed that CSP is restricted to presynaptic termini (Zinsmaier et al., 1990; Kohan et al., 1995). The vertebrate homolog of CSP was identified in *Torpedo californica* electric organ (Gundersen and Umbach, 1992). Three mammalian homologs of the single fly CSP gene have been identified—CSPα, CSPβ, and CSPγ (Fernández-Chacón et al., 2004). CSPα is the functional homolog of the fly CSP and is localized to synaptic vesicles at the presynaptic terminal. CSPβ is expressed in both auditory hair cell neurons and testes, while CSPγ is expressed solely in testes (Fernández-Chacón et al., 2004; Schmitz et al., 2006).

CSPα has the domain organization of a DNAJ C-class Hsp40 co-chaperone. It possesses an N-terminal helix (residues 1–13 in rat), followed by a J domain (residues 14–81), an adjacent linker (residues 82–111), and an eponymous cysteine string domain containing 13–15 extensively palmitoylated cysteine residues in a 25-residue motif (residues 112–136; Braun and Scheller, 1995; Patel et al., 2016), which is immediately adjacent a palmitoylacyltransferase (PAT) recognition site responsible for palmitoylation of the protein (residues 136–145). CSPα also possesses a C-terminal domain (residues 146–198; Figure 2C).

Through its J domain, CSPα interacts with Hsc70 (Braun et al., 1996). Within alpha helices 1 and 2 of the 4-alpha helix J domain, there is an HPD motif that, when mutated, abolishes CSPα's stimulation of Hsc70 ATPase activity (Chamberlain and Burgoyne, 1997). Yeast two-hybrid studies showed that the cysteine string region may also be responsible for an interaction between CSPα and the small glutamine-rich tetratricopeptide repeat-containing protein (SGT; Tobaben et al., 2003). CSPα recruits SGT and Hsc70 to synaptic vesicles where its interaction leads to maximal Hsc70 ATPase activity. However, a recent study demonstrated the role for the Hsc70/SGT interaction in stimulating protein folding, while inhibiting Hsc70-mediated synaptic membrane deformation for autophagy of Hsc70 clients (Uytterhoeven et al., 2015). Hence, the physiological relevance of the Hsc70/CSPα vs. Hsc70/CSPα/SGT complex still needs to be resolved.

The N-terminal domain of CSPα is phosphorylated by protein kinase A on a serine at position 10 (phospho-Ser^10^), effectively reducing accessibility to the α1 helix of the N terminus and inhibiting binding to putative clients, namely syntaxin and synaptotagmin I (Figure 2B). However, this does not alter the J domain, HPD motif, or interactions with Hsc70 (Patel et al., 2016). CSPα also has a linker between the cysteine string and J domains that may have an effect on the interaction between CSPα and clients, however this remains to be established. Deletion and point mutations within this region should elucidate the most important residues, and give additional insight into its interactions with client proteins.

CSPα is one of the most heavily palmitoylated proteins known. In the brain, the CSPα cysteine string domain is normally fully palmitoylated by several PATs, most prominently DHHC5/HIP14 (Ohyama et al., 2007; Stowers and Isacoff, 2007). The palmitoylation of cysteine residues in the cysteine string allows for peripheral membrane association and is essential for targeting CSPα to synaptic vesicles. On average, there are 2.8 copies of CSPα per synaptic vesicle (Takamori et al., 2006). CSPα is also found on secretory vesicles in non-neuronal tissues.

Most of our present understanding of CSPα function comes from knockout (KO) studies in flies and mice. In *Drosophila*, CSP nulls result in 95% embryonic lethality, but those flies that do survive to adulthood exhibit progressive sluggishness, spasmic jumping, shaking, uncoordinated movement, and high-temperature paralysis, as well as premature death (Zinsmaier et al., 1990, 1994; Burgoyne and Morgan, 2015). These phenotypes were ascribed to defects in Ca^2+^ dynamics, neurotransmission deficits, progressive deterioration of CSPα null synapses and eventual neurodegeneration, all phenomena that may be explained by the accumulation of incorrectly folded client proteins at presynaptic termini leading to dysfunction in neurotransmitter release (Umbach et al., 1994; Zinsmaier et al., 1994). Mutations in both *Drosophila* CSP and Hsc70 cause a similar temperature-sensitive loss of evoked neurotransmitter release that can be restored by elevating Ca^2+^ levels (Bronk et al., 2001). These common phenotypes are consistent with the idea that Hsc70 and CSP function together to chaperone the synaptic vesicle fusion machinery. Overexpression of *Drosophila* CSP suppresses the decrease of evoked release induced by the overexpression of syntaxin 1A, suggesting that CSP modulates protein-protein interactions of syntaxin, including SNAREs (Nie et al., 1999). In addition, CSP interacts with the SNARE synaptobrevin and the Ca^2+^ sensor synaptotagmin in a phosphorylation dependent manner (Evans and Morgan, 2002; Boal et al., 2004).

CSPα KO mice are normal at birth but exhibit a progressive sensorimotor phenotype and age-dependent synapse deterioration beginning around P20 (Fernández-Chacón et al., 2004). These mice also exhibit degeneration of retinal photoreceptors and an increased susceptibility for degeneration of the most active GABAergic synapses, followed by death around 8 weeks (Schmitz et al., 2006; García-Junco-Clemente et al., 2010). Congruently, hippocampal neuron cultures from CSPα KO mice show selective vulnerability of synaptotagmin-2^+^ GABAergic cells to neurodegeneration, as well as mIPSC (decreased frequency), and mEPSC (decreased amplitude) neurotransmission deficits (García-Junco-Clemente et al., 2010). CSPα also functions in motor neurons at neuromuscular junctions to maintain the readily releasable pool of vesicles (Rozas et al., 2012). In CSPα KO mice, a defect in vesicle recycling at the neuromuscular junction may be partially responsible for the canonical motor phenotypes observed in these animals. Combined, these studies indicate that higher neuronal activity leads to faster synaptic deterioration and neurodegeneration in the absence of CSPα.

Detailed electrophysiological analysis of P10 CSPα KO pups prior to synapse loss and neurodegeneration has revealed normal Ca^2+^ currents and neurotransmission (Fernández-Chacón et al., 2004), suggesting that CSPα is not directly required for neurotransmitter release. At P20-P30, evoked release becomes asynchronous and deteriorates progressively with age, indicating that CSPα clients play key roles in the maintenance of synaptic structure and function (Fernández-Chacón et al., 2004). Congruently, Rozas and colleagues have demonstrated the importance of CSPα in synaptic vesicle exo- and endocytosis; CSPα KOs show decreases in exocytic release sites, as well as endocytosis. CSPα KO synapses do not efficiently recycle membrane back to generate synaptic vesicles (Rozas et al., 2012). This leads to decreases in the levels of releasable vesicles, further impairing the neuron's exocytic capacity.

Interestingly, genetic studies have demonstrated that CSPα KO phenotypes are rescued by α-synuclein overexpression (Chandra et al., 2005), a key gene in the pathophysiology of Parkinson's disease, while α-synuclein KO exacerbates these phenotypes. These genetic crosses suggest that synucleins act downstream of CSPα to stabilize one or more of its clients.

In order to ascribe mouse CSPα KO phenotypes to molecular processes, there has been a concerted effort to identify CSPα clients, mainly through two approaches—binding assays and quantitative proteomics. Via binding assays, SNAP-25, the t-SNARE for synaptic vesicle fusion, was identified as the first CSPα client (Chandra et al., 2005; Sharma et al., 2011). SNAP-25 was confirmed as a client through analysis of CSPα KO brains. These brains exhibit significant decreases in SNAP-25 levels due to its activity-dependent ubiquitination and degradation (Sharma et al., 2011). Interestingly, SNAP-25 directly binds Hsc70 as opposed to binding via CSPα.

To find additional CSPα clients, our lab embarked on a proteomic screen (Zhang et al., 2012). The rationale for the screen design was that clients of CSPα would be misfolded and targeted for degradation in the absence of CSPα, leading to decreases in their levels in CSPα KO brains. Thus, comparing the synaptic proteome of wild-type and CSPα KO would be instructive in identifying clients in an unbiased and systematic manner (Zhang et al., 2012). Through this screen we identified 22 proteins, other than chaperones, that were decreased in the CSPα KO proteome. This list included SNAP-25, affirming our screen design. We validated many of these proteins by orthogonal methods. Through direct binding in the presence of ADP, we showed that the endocytic GTPase dynamin-1 is also a client of CSPα (Zhang et al., 2012). Furthermore, addition of purified dynamin-1 results in increases in Hsc70 ATPase activity in the presence of CSPα, confirming it as a Hsc70/CSPα client. The oligomerization of dynamin-1 is deficient in CSPα KO brains, implying either dynamin-1 self-assembly or disassembly is regulated by CSPα.

Through direct binding and mass spectrometric approaches, we confirmed that SNAP-25 and dynamin-1 are bona-fide CSPα clients and showed that CSPα has in the range of 5–6 clients. Previously, other known CSPα client proteins include VAMP-1, G-protein subunit, and N-type Ca^2+^ channels (Chamberlain et al., 2001).

CSPα KO phenotypes are likely to be a compound loss- or gain-of-function of CSPα clients. For instance, SNAP-25 knockdown in CSPα KO animals exacerbates the CSPα phenotype, while lentiviral expression of SNAP-25 in CSPα KO mice rescues the neurodegenerative phenotype (Sharma et al., 2012). These data suggest that loss of SNAP-25 function in CSPα KOs results in the synaptic phenotypes and neurodegeneration associated with CSPα KO or dysfunction. These results are consistent with its function in maintenance of synaptic exocytic machinery. Intriguingly, despite the ability of overexpressed α-synuclein to rescue CSPα phenotypes, α-synuclein does not rescue SNAP-25 levels, only SNARE complex levels, indicating that α-synuclein rescue is not mediated by SNAP-25. In the case of dynamin-1, rescue experiments have not been completed to determine its mode of action in CSPα KOs. Identification of CSPα's clients has revealed that neurodegeneration in CSPα mutants and KOs may be due to an exo-endocytosis coupling defect, resulting in the neuron's failure to maintain and recycle synaptic vesicles during prolonged stimulation.

## DNAJC6: auxilin

Auxilin is the best studied mammalian Hsp40 and was identified in a study of the clathrin-coated vesicle (CCV) uncoating mechanism as a cofactor for Hsc70/Hsp70c (Ungewickell et al., 1995). It is unique among the Hsp40 co-chaperones in that it has only one known client, although research is underway to elucidate its influence on other coat proteins, such as COPII (Ding et al., 2016).

Auxilin is a brain-specific, 970-amino acid protein that is enriched at presynaptic termini. Auxilin has a C-terminal J domain (residues 814–910) that allows for its interaction with Hsc70 (Figure 2C; PDB: 3N0A). The auxilin J domain highly divergent, with an extra N-terminal alpha helix as well as an extended loop between the first and second helices of the domain that is important for Hsc70 binding (Jiang et al., 2003). Deletions within auxilin's J domain inhibit its Hsc70 co-chaperone activity, but not its interaction with Hsc70, congruent with its divergent structure (Holstein et al., 1996). Auxilin also has a lipid binding domain (residues 60–387), which contains a PTEN phosphatase-like domain followed by a C2 domain (Guan et al., 2010). *In vitro* studies have shown that the auxilin C2 domain binds specifically to phosphoinositide-containing membranes, and the PTEN domain has affinity to phosphoinositides PI4P and PIP_2_. Together, this region is required for recruitment of auxilin to clathrin-coated vesicles following dynamin mediated scission (Guan et al., 2010). In addition, auxilin has a clathrin binding domain (residues 547–814) that facilitates interaction with its client (Haynie and Ponting, 1996; Edvardson et al., 2012). Proper auxilin function necessitates physical association of the clathrin binding domain and the J-domain, as the combination of separate J domain deletion mutants and clathrin binding domain mutants does not rescue the clathrin coated vesicle accumulation phenotype of auxilin KOs (described below).

**Figure 3:**
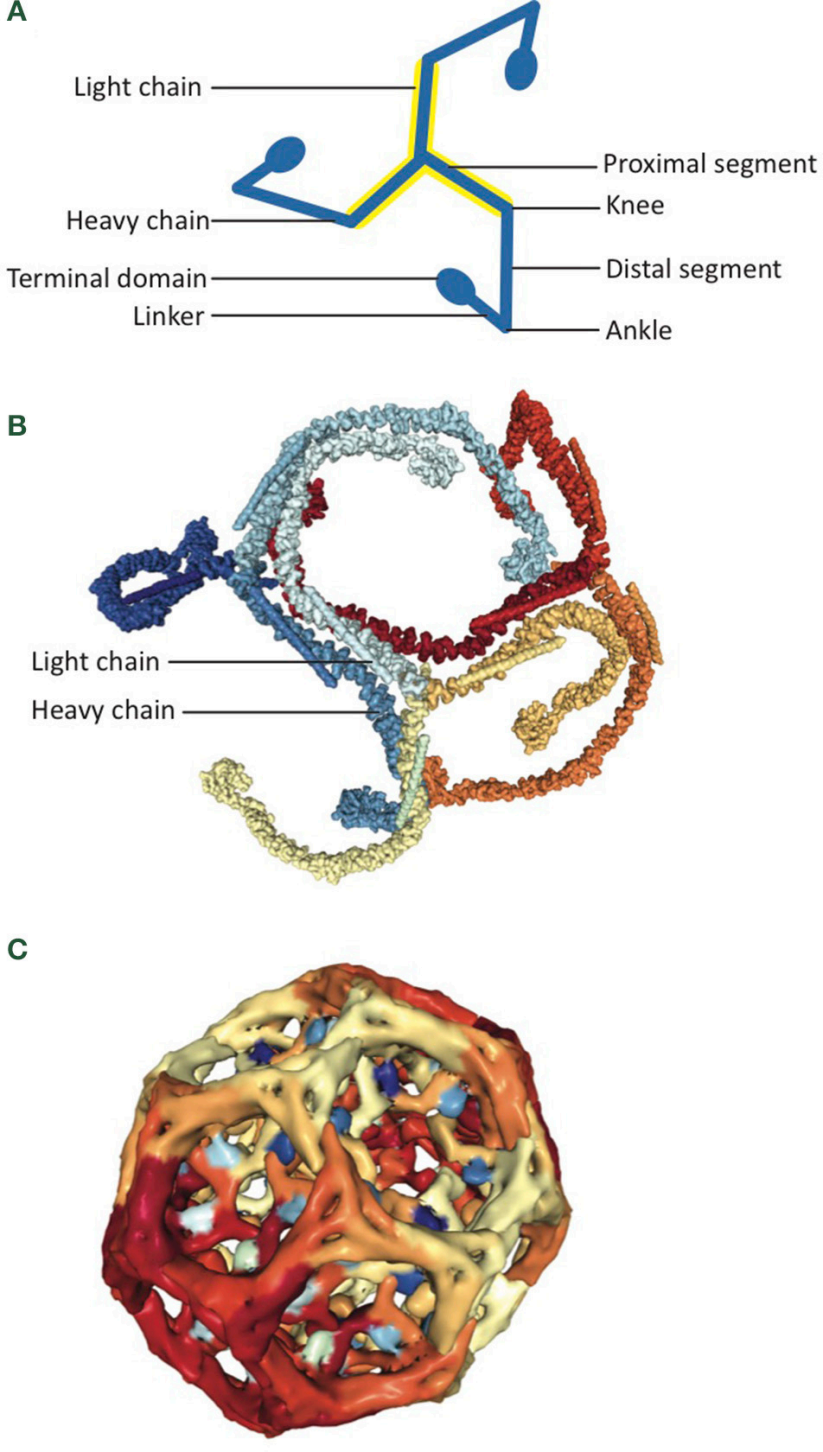
**Auxilin is an Hsc70 co-chaperone for clathrin uncoating. (A)** Cartoon depicts structure of a clathrin triskelion. Three clathrin ankle segments cross to form a stable clathrin cage. **(B)** Assembled clathrin coat containing clathrin heavy chain and clathrin light chain, as determined by electron microscopy (PDB:3IYV; Fotin et al., 2004b). **(C)** The J domain of auxilin (shades of blue) interacts with the clathrin coat (shades of red and yellow; PDB: 1XI5; Fotin et al., 2004a).

The structure of auxilin bound to the clathrin coat has been resolved by electron microscopy and has provided great insight into the mechanisms of auxilin-dependent clathrin uncoating. Auxilin was shown to bind clathrin at a 1:3 ratio at the clathrin ankle segment. Ankles from three clathrin triskelia are typically crossed in a stable clathrin coat (Figure 3), and auxilin binding causes a shift in ankle orientation that leads to a disruption of coat structure (Fotin et al., 2004b). Auxilin bound to the clathrin ankle recruits Hsc70 to the vertices of the clathrin coat, where it hydrolyzes ATP and binds clathrin, further increasing the strain on clathrin cage interactions (Fotin et al., 2004b; Xing et al., 2010).

Functional studies have established that auxilin and Hsc70 interact in the context of clathrin-mediated endocytosis to disassemble clathrin coats (Ungewickell et al., 1995). As a classical Hsp40, the presence of auxilin increases Hsc70 ATPase activity 5-fold (Jiang et al., 1997). Auxilin binds clathrin independently of nucleotide, initiating a strain on the clathrin coat structure, and then recruits Hsc70 which allows for further disruption of the clathrin cage, ultimately resulting in its disassembly (Fotin et al., 2004b; Xing et al., 2010). Auxilin has a high affinity for Hsc70-ATP, while clathrin has a high affinity for Hsc70-ADP (Jiang et al., 1997). When ATP hydrolysis occurs, auxilin releases Hsc70 and clathrin, but Hsc70 remains bound to clathrin, possibly preventing its re-oligomerization (see Figure 1; Holstein et al., 1996). The exact ratio of this reaction *in vivo* is somewhat controversial. Researchers have demonstrated a 1:3:3 ratio of Auxilin:Hsc70: clathrin, as well as a 1:1:>3 ratio (Böcking et al., 2011, 2014). There may be a variety of Auxilin:Hsc70:clathrin ratios dependent on cellular conditions and bond stability, and lower ratios may be possible but energetically unfavorable.

Auxilin KO in mice results in decreased viability and birth weight in a copy number-dependent manner (Yim et al., 2010). In auxilin KO animals, the efficiency of clathrin uncoating is impaired, which leads to an excess of clathrin-coated vesicles at the synapse and impairments in endocytosis (Yim et al., 2010). While auxilin is brain-specific, its homolog, G-associated kinase (GAK; DNAJC26), is broadly expressed and may partially compensate for a loss of auxilin function (Yim et al., 2010) as levels of GAK increase in auxilin KOs. Furthermore, Lee et al. demonstrated that conditional deletion of GAK in adult mice is lethal, demonstrating the essential role of clathrin-mediated endocytosis in all cell types (Lee et al., 2008). Surprisingly, auxilin/GAK double KO with expression of only the clathrin binding and J domains can rescue lethality despite the mice having a body size similar to auxilin KO alone, further confirming that auxilin defects are due to reduced clathrin uncoating and vesicle recycling (Park et al., 2015).

While clathrin cages have been long thought to be the only clients of auxilin, recent data suggest that it functions early in the secretory pathway on COPII vesicles (Ding et al., 2016). In cells depleted of auxilin, trafficking is disrupted in COPII-dependent regions between the ER and Golgi, as well as throughout the Golgi, and COPII-mediated vesicle fusion is disrupted. This finding has raised the intriguing possibility that auxilin has additional clients.

## DNAJC13: receptor mediated endocytosis 8

RME-8 is a 2,000 amino acid protein that was identified in a *C. elegans* screen for organisms defective in the endocytosis of yolk proteins (Zhang et al., 2001) and is required for *C. elegans* survival and development (Fujibayashi et al., 2008). It is expressed in all tissues in varying levels (Ishikawa et al., 1998). RME-8 has a J domain (residues 1,301–1,366) toward the C-terminal half of the protein (Figure 2C). Through its J domain, RME-8 interacts and specifically stimulates the ATPase activity of Hsc70-4, with no effect on any other Hsc70 family member (Chang et al., 2004). Flanking its J domain, RME-8 contains four repeats of about 90 amino acids known as IWN repeats, which are conserved among RME-8 homologs but have an as-of-yet undetermined function (Zhang et al., 2001; Chang et al., 2004; Girard et al., 2005; Fujibayashi et al., 2008; Xhabija and Vacratsis, 2015). RME-8 also contains a pleckstrin homology domain (residues 312–350) that allows it to interact with the phosphoinositide PI3P, which aids in the localization of RME-8 to PI3P rich membranes as in endosomes (Xhabija and Vacratsis, 2015).

RME-8 is a peripheral membrane protein localized to early, but not late, endosomes where it appears to associate with the membrane via the PI3P/pleckstrin homology domain (Fujibayashi et al., 2008). In *C. elegans*, RME-8 is required for receptor mediated endocytosis as well as fluid tracer uptake (Chang et al., 2004). Although RME-8 is required for invertebrate survival, its knockdown has minimal effects on endocytosis in mammalian cultured cells. However, C-terminal deletion (lacking the last IWN repeat, but with the J domain) leads to formation of RME-8 puncta and vacuoles, accumulation of ubiquitinated proteins, and changes in early endocytic morphology (Fujibayashi et al., 2008). This defect suggests that the C-terminus of RME-8 plays an integral role in trafficking through early endosomes to late and recycling endosomes. Congruent with such a function, RME-8 is localized to Rab5 positive organelles throughout the cell in mammalian neurons, including synapses.

Two roles have been described for RME-8 in trafficking through early endosomes. First, similar to its role in *C. elegans*, RME-8 is important in clathrin-mediated endocytosis in *Drosophila*, and is essential for the uptake and internalization of membrane ligands and receptors (Chang et al., 2004). Defects in RME-8 in *Drosophila* lead to defects in the uptake of endosomal tracers, as well as disorganization of the endosomal compartment. These phenotypes of RME-8 mutants bear a strong resemblance to mutations in Hsc70-4, suggesting that the two genes act in a common pathway. Additionally, studies in HeLa cells have demonstrated that siRNA-mediated knockdown of RME-8 leads to defects in EGF uptake into the cells (Girard et al., 2005). However, as RME-8 has no clathrin binding site, it must modulate clathrin-mediated endocytosis through a mechanism independent of auxilin. This suggests that RME-8 may exert its effects between the dynamin-1 and auxilin stages of endocytosis, as is has similarities to both. Second, RME-8 has been shown to be a component of the retromer complex, which executes sorting and retrieval to the trans-Golgi (Girard et al., 2005; Freeman et al., 2014; Seaman and Freeman, 2014). RME-8 aids in localization of the WASH (WASP and Scar homolog) complex to endosomal tubules for retromer-mediated endosomal protein sorting (Perrett et al., 2015). Currently, a thorough mechanism of RME-8 function in the early endocytic pathway is lacking, and identification of additional clients, interactors, and members of the pathway will provide detail on RME-8's role in endocytosis.

## Disaggregase: Hsc70, Hsp110, DNAJA1, and DNAJB1

Metazoans lack the key Hsp100 disaggregases, such as the yeast Hsp104, which exist in all non-metazoans. Recently, the metazoan disaggregase was identified and shown to consist of Hsc70, Hsp110, and DNAJA and B proteins (Nillegoda et al., 2015). This disaggregase complex functions in the disaggregation of insoluble proteins, including those in amyloid-like structures. The transcription of Hsp110 is stimulated by conditions of stress, and it works in concert with other quality control proteins to restore native protein folding states (Zuo et al., 2016).

Hsp110 was identified as an Hsp70 interactor and NEF (ATP for ADP) to induce client release from Hsc70 (Dragovic et al., 2006). Though Hsp110 on its own possesses chaperone activity *in vitro*, it acts only as a NEF in the presence of Hsc70 (Mattoo et al., 2013).

Several new studies have demonstrated the disaggregative properties of Hsp110 with both model clients such as aggregated luciferase and most significantly with neurodegenerative disease-linked proteins such as α-synuclein and prion protein (Gao et al., 2015; O'Driscoll et al., 2015). Furthermore, studies have demonstrated Hsp110's ability to prevent aggregation-prone proteins from becoming toxic to cells (Eroglu et al., 2010). As a disaggregase, Hsp110 can work with both DNAJA1 and DNAJB1 in conjunction with Hsc70 to disaggregate and refold aggregated proteins (Nillegoda et al., 2015). In the case of the Parkinson's disease linked α-synuclein protein, Hsc70, DNAJB1 and Hsp110 individually have little effect on insoluble α-synuclein fibrils (Gao et al., 2015). However, when the three chaperone proteins are combined *in vitro*, they selectively disaggregate and solubilize α-synuclein fibrils into small polymers and monomers in a concentration-dependent manner. By contrast, DNAJA family members do not effectively resolubilize α-synuclein fibrils, demonstrating the selectivity of DNAJB proteins in this interaction.

This newly discovered disaggregase activity is consistent with prior findings demonstrating that Hsp110 regulates aggregation in several neurodegenerative diseases. Hsp110 mouse KOs show increased tau phosphorylation and amyloid beta accumulation in the brain (Eroglu et al., 2010). Hsp110 also interacts with Hsc70c and DNAJB1 to protect *Drosophila* rhabdomeres against the accumulation and toxicity of polyQ expansion proteins (Kuo et al., 2013). The disaggregase also prevented neurodegeneration caused by polyQ proteins. Furthermore, DNAJB1 and Hsp70 family member HspA1A can function in concert to reduce the levels of aggregated huntingtin (Rujano et al., 2007). Importantly, transgenic overexpression of Hsp110 in SOD1 mutant mice rescues their survival, further highlighting the importance of functional disaggregation machinery in combating misfolding and neurodegenerative diseases *in vivo* (Nagy et al., 2016).

The above data raise the exciting possibility that the canonical protein aggregation occurring in age-related neurodegenerative diseases may be a reversible with appropriate disaggregase chaperone activity. Aggregation of proteins is known to have both loss-of-function mechanisms, due to misfolding and decreased normal activity, and toxic gain-of-function mechanisms, such as the sequestration of neural and synaptic chaperones. If protein aggregates can be disassembled, both of these toxic mechanisms can be prevented. In the future, disaggregase chaperones such as Hsp110 may be a broad-acting therapeutic target for the protein aggregates that occur in a variety of neurodegenerative diseases, most prominently Alzheimer's and Parkinson's disease (Shorter, 2016).

## Synaptic co-chaperones and neurodegenerative diseases

Mutations in synaptic Hsp40 co-chaperones are causally related to human neurodegenerative diseases, underscoring the importance of the synaptic proteostasis network for the healthy brain. In CSPα, two mutations in the cysteine string region (L115R and L116Δ) result in autosomal dominant adult neuronal ceroid lipofuscinosis (ANCL), also known as Kufs disease and Parry disease (Benitez et al., 2011; Nosková et al., 2011; Velinov et al., 2012; Cadieux-Dion et al., 2013). ANCL is a hereditary, adult-onset, progressive neurodegenerative disease with variable clinical symptoms. Clinically, patients present with epilepsy, movement disorders, dementia, anxiety, speech changes, and early mortality (Burgoyne and Morgan, 2015). Pathologically, ANCL is characterized by intralysosomal accumulation of lipofuscin containing the protein saposin. ANCL patient brains show decreased CSPα protein levels as well as the presence of CSPα aggregates (Nosková et al., 2011), though this was not evident in early stages of the disease (Benitez et al., 2015). These proteostatic changes can be recapitulated by expression of L115R and L116Δ mutant CSPα in mouse CSPα KO neurons. This leads to overall low CSPα levels, as well as accumulation of the mutant CSPα in the cell body and low expression at synapses. This suggests that the mechanism of neurodegeneration in ANCL patients may be both a loss-of-function at the synapse, and an aberrant gain-of-function at the cell body.

Biochemical characterization of the L115R and L116Δ CSPα mutants revealed that they function as co-chaperones, consistent with the mutations being in the cysteine string domain and not in the J- or C-terminal domains (Zhang and Chandra, 2014). However, both ANCL mutants (L115R and L116Δ) have a high propensity to oligomerize and form aggregates *in vitro*, with mutant CSPα forming ubiquitinated inclusions (Zhang and Chandra, 2014). Presently, it is controversial whether the aggregates of ANCL mutant CSPα are palmitoylated, with contradictory data available (Greaves et al., 2012; Zhang and Chandra, 2014; Diez-Ardanuy et al., 2017). Regardless, ANCL mutant CSPα can readily co-oligomerize with the wildtype CSPα protein, possibly explaining the autosomal dominant nature of this disease. Significantly, oligomerization (both homo- and hetero-) leads to decreased co-chaperone activity, suggesting a dominant negative mechanism of mutant CSPα, as the protein present in higher molecular weight species cannot function as a co-chaperone for Hsc70 (Zhang and Chandra, 2014).

Remarkably, analysis of L115R and L116Δ ANCL patient brains revealed increased expression, decreased specific activity, and mislocalization of palmitoyl protein thioesterase 1 (PPT1), the enzyme responsible for the removal of palmitoyl groups from proteins, including CSPα (Henderson et al., 2016). Loss-of-function mutations in PPT1 also cause NCL (Vesa et al., 1995), suggesting that the mutations in the cysteine string region of the protein affect CSPα's palmitoylation status, even though the residual mutant CSPα appears fully palmitoylated. This hypothesis is supported by a novel CSPα ANCL mutation resulting in duplication of a segment of the cysteine string region (Jedličková et al., 2016). However, further study is required to understand the mechanism by which CSPα affects PPT1 activity, or vice versa, and PPT1's role in proteostasis. Studies of mutant CSPα palmitoylation and membrane association may provide more insight into the nature of this interaction.

CSPα dysfunction may also be related to other neurodegenerative diseases. CSPα levels have been shown to decrease in the degenerating regions of Alzheimer's disease patient brains (Zhang et al., 2012). Impaired assembly of the SNARE complex, a hallmark of CSPα KO brains, has also been identified in Alzheimer's and Parkinson's diseases (Garcia-Reitböck et al., 2010). Recently, Sambri and colleagues demonstrated decreased CSPα levels in a mouse model of mucopolysaccharidosis type IIIA, a lysosomal storage disease. Importantly, overexpression of CSPα rescued many of the phenotypes in the mucopolysaccharidosis mouse model (Sambri et al., 2017), supporting a functional role for CSPα in this disease. The present idea is that in mucopolysaccharidosis type IIIA, CSPα-mediated lysosomal dysfunction may disrupt the activity of the presynaptic compartment through dysregulation of SNARE proteins.

Auxilin and RME-8 mutations are associated with Parkinson's disease in humans. Loss-of-function mutations in auxilin cause juvenile onset (between 10 and 21 years old) Parkinson's disease. This form of Parkinson's disease is characterized by visual hallucinations, cognitive deterioration, epilepsy, and psychosis, as well as the typical motor symptoms of tremor, rigidity, and bradykinesia (Köroğlu et al., 2013; Elsayed et al., 2016). In a study of an inbred family with high rates of juvenile parkinsonism, Köroğlu et al. identified a combination homozygous nonsense mutation and missense mutation in the DNAJC6 gene that resulted in premature truncation of the auxilin protein (Köroğlu et al., 2013). The nonsense mutation is the result of a C to T substitution at position 2200 of the DNA sequence, resulting in a stop codon within exon 16 of the mRNA and truncation of about 1/5 of the C-terminal portion of auxilin (p.Q734X), including the J-domain. The missense mutation results in the substitution of cysteine at position 61 for serine, a change that is likely deleterious to protein structure and function. Additionally, several other mutations have been identified in auxilin that are linked to juvenile onset Parkinson's disease (Table 2). Milder mutations in auxilin lead to later onset, while complete loss-of-function leads to earlier onset forms (Elsayed et al., 2016). Congruently, sequence variants in GAK, the auxilin homolog, have also been linked to Parkinson's disease risk by meta-analysis of GWAS, as well as in a study of sporadic Parkinson's disease in China (Nalls et al., 2014; Zhang et al., 2016). It is presently not clear why clathrin uncoating defects through the loss-of-function of auxilin should lead to Parkinson's disease. One possibility is that deficiency leads to alternative modes of endocytosis that is taxing to dopaminergic neurons. Another possibility is that Hsc70 is sequestered to CCVs leading to a severe synaptic proteostasis defect. Alternatively, auxilin has additional clients in substantia nigra neurons.

**Table 2:** **Mutations in auxilin (DNAJC6) associated with Parkinson's disease**.

**Mutation**	**Zygosity**	**Protein**	**References**
c.801-2 A>G	Homozygote	-Deletion of aa 268-328 -premature stop	Edvardson et al., 2012
**c.1468** + **83 del**	Compound heterozygote		Olgiati et al., 2016
c.2200C>T	Homozygote	p.Q734X	Köroğlu et al., 2013
**c.2038** + **3 A**>**G**	Compound heterozygote		Olgiati et al., 2016
c.2223A>T	Homozygote	p.Thr741^*^	Olgiati et al., 2016
c.2365C>T	Homozygote	p.Gln789^*^	Elsayed et al., 2016
c.2371C>T		p.Gln791^*^	
c.2779A>G	Homozygote	p.Arg927Gly	Olgiati et al., 2016

*Bold denotes mutations associated with double heterozygote forms of Parkinson's disease*.

* *Denotes premature stop codon*.

A mutation in RME-8 was identified in a large pedigree with autosomal dominant Parkinson's disease (p.Q734X). In this Mennonite family, a heterozygous N855S mutation in RME-8 was discovered by exome sequencing of the proband and relatives with Parkinson's disease (Vilariño-Güell et al., 2014). The mutation falls between the first two IWN domains (Zhang et al., 2001). However, the N855S mutation was not always associated with disease in this pedigree. A recent publication using the same family found a mutation in an uncharacterized ORF encoding a protein TMEM230, which is localized to synapses, using positional mapping (Deng et al., 2016). This new study casts the role of RME-8 in Parkinson's disease in doubt and requires further examination to identify the contribution of each protein to the disease.

**Figure 4:**
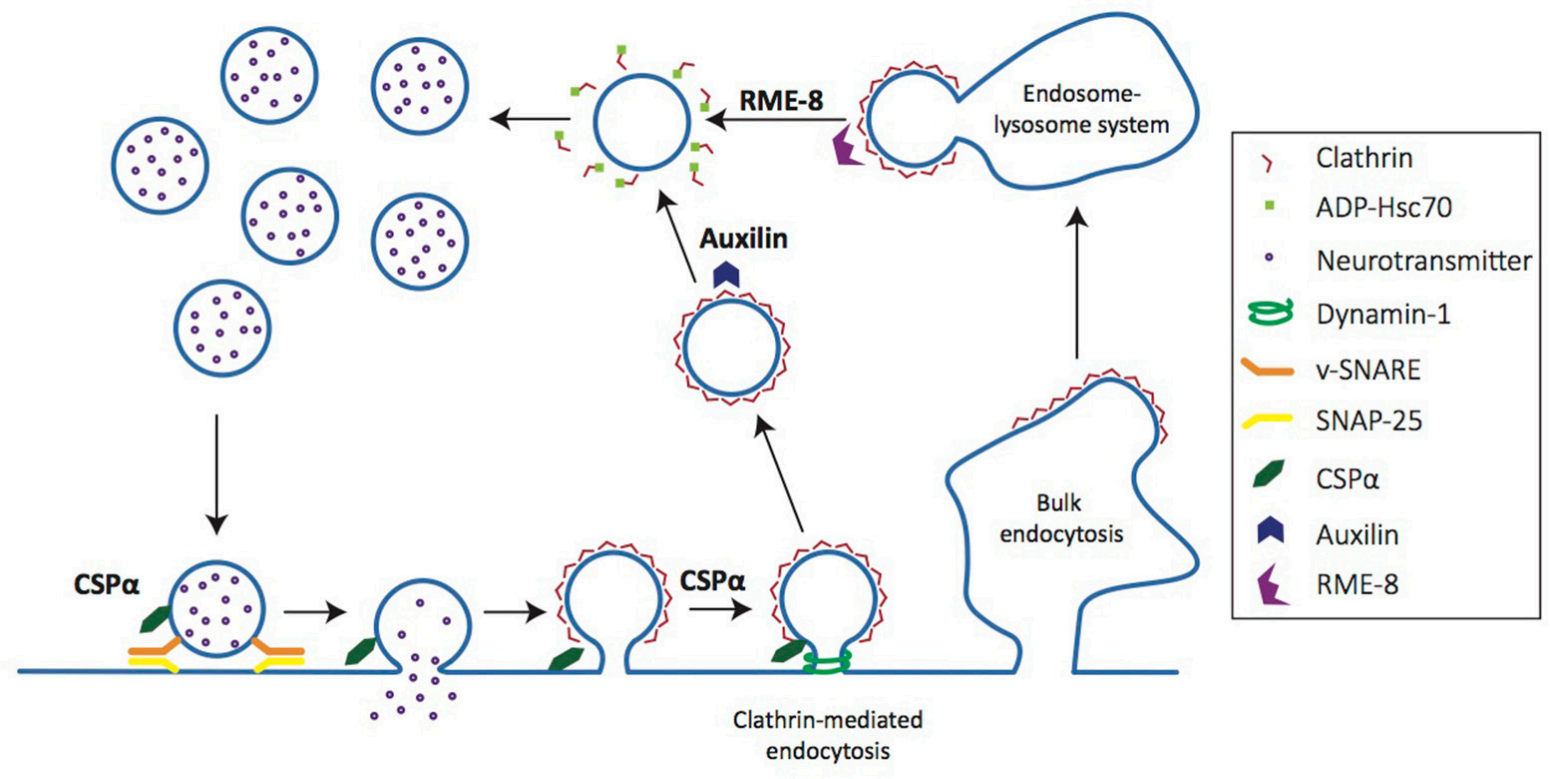
**Co-chaperones regulate distinct steps of the synaptic vesicle cycle**. Cartoon depicts synaptic vesicle cycle. CSPα chaperones SNAP-25, a t-SNARE involved in vesicle fusion for neurotransmitter release. CSPα also chaperones dynamin-1, a GTPase necessary for vesicle scission in clathrin-mediated endocytosis. Auxilin acts on clathrin-coated vesicles, uncoating them to generate nascent vesicles that are returned to the recycling pool. RME-8 regulates clathrin uncoating at the early endosome.

Clients of CSPα, auxilin, and RME-8 all participate in either synaptic vesicle exocytosis, endocytosis and/or recycling of synaptic vesicles and membrane components (Figure 4). Thus, the association of these co-chaperones with neurodegenerative diseases highlights the importance of efficient functional synaptic exocytic and endocytic machinery in synapse- and neuroprotection. In particular, the synaptic vesicle endocytosis deficits seen in co-chaperone mutants raise an important question: when clathrin mediated endocytosis is impaired, do the alternative modes of synaptic vesicle cycling that ensue lead to protein sorting inefficiencies that further disrupt synaptic proteostasis and cause synaptic dysfunction and loss? Hence, understanding synaptic vesicle cycling in co-chaperone mutants will be very instructive to understanding early steps in neurodegeneration.

## Synaptic proteostasis, neurodegeneration, and aging

It is well established that Aβ in Alzheimer's disease and α-synuclein in Parkinson's disease aggregate to form defining pathologies—amyloid plaques and Lewy bodies, respectively. Recent evidence suggests that their aggregation begins at the synapse and/or that synapses are sites of action of these pathological aggregates. In line with this idea, aggregation of α-synuclein has been demonstrated to begin at synaptic termini where the protein normally resides (Kramer and Schulz-Schaeffer, 2007; Scott et al., 2010; Spinelli et al., 2014), even though Lewy bodies are present in soma. These synaptic aggregates are likely to contribute to the synaptic dysfunction and synapse loss observed in Parkinson's disease (Nemani et al., 2010; Scott and Roy, 2012). Therefore, there is new interest in understanding why the synaptic proteostasis network is unable to deal with neurodegenerative disease aggregates and is overwhelmed during disease processes. One possible explanation is that the accumulation of disease-related proteins can not only cause a toxic gain-of-function, but may lead to a loss-of-function of specific proteins at the synapse through the sequestration of bystander proteins, especially chaperones (Rampelt et al., 2012). For example, aggregation of expanded polyglutamine repeat proteins such as those implicated in Huntington's disease and spinocerebellar ataxia can inhibit clathrin-mediated endocytosis by competitive binding and sequestration of Hsc70, and decreased Hsc70 expression (Yamanaka et al., 2008; Yu et al., 2014).

Age is the single biggest risk factor for neurodegenerative disease. New studies examining the reason behind this have uncovered several probable links to changes in the synaptic proteostasis network (Labbadia and Morimoto, 2015). Analysis of the human chaperome of distinct brain regions showed that Hsp60s, Hsp40s, and Hsp70s were consistently repressed with age. Among repressed genes, the Hsp40s exhibited the most significant change, with 62% of 48 Hsp40 genes repressed in aging. Pertinent to synaptic proteostasis, DNAJC5 and DNAJA1 levels are also decreased with aging (Brehme et al., 2014) and in Alzheimer's disease (Zhang et al., 2012). This analysis of the chaperome in aged human brain revealed a concordant exacerbation of responses in neurodegenerative disease, and provides evidence for similar changes in the synaptic proteostasis network in both aging and neurodegenerative disease. The causal relationship between age-related decrement in proteostasis and protein aggregation has been examined in worm. In aging *C. elegans*, dysregulation of proteostasis has been demonstrated to lead to the accumulation of aggregation-prone proteins, thus starting a vicious cycle (Walther et al., 2015). From a therapeutic standpoint, in *C. elegans*, the demonstrated correlation between age and decreased synaptic integrity can be prevented by increased expression of the master heat shock transcription factor *hsf*-1 (Toth et al., 2012).

## Future directions

While recent years have seen progress in understanding the role of synaptic chaperone systems in maintaining functional synaptic connections, more research is required to deepen our understanding of synaptic chaperone clients, the relationship between changes in synaptic proteostasis and normal aging as well as neurodegenerative diseases.

In the case of CSPα, a complete characterization of its clients will help delineate their importance to CSPα KO phenotypes. This analysis can lead to testable hypothesis as to why α-synuclein rescues CSPα KO phenotypes. It is presently unclear how ANCL mutations disrupt CSPα co-chaperone activity and what role palmitoylation plays in this disruption *in vivo* due to the paucity of animal models. Although auxilin has been well-characterized, recent evidence of its functions in alternative endocytic pathways prompts further mechanistic study. As COPII is only the second client of auxilin identified, further effort on unearthing additional clients may identify new roles for auxilin's co-chaperone activity. For Parkinson's disease linked to auxilin mutations, it remains to be investigated why clathrin-mediated endocytosis deficits leads to neurodegeneration. Additionally, RME-8 is the least-studied co-chaperone discussed in this review. The clients of RME-8 need to be identified, and this will address the mechanisms by which RME-8 regulates endocytosis at the synapse. Studies have already made it clear that RME-8 plays a distinct role from auxilin, but its specific function in clathrin-mediated endocytosis requires more investigation. Finally, additional investigation is required to identify the effect of RME-8 in familial Parkinson's disease, and to determine whether its dysfunction works independently or synergistically with the mutation in TMEM230 to influence disease progression.

## Conclusions

The importance of co-chaperones in the maintenance of synapse function and the nervous system is becoming clear given that their dysfunction leads to loss of synapses and neurodegenerative disease.

## Author contributions

ELG and SSC prepared the manuscript.

### Conflict of interest statement

The authors declare that the research was conducted in the absence of any commercial or financial relationships that could be construed as a potential conflict of interest.
